# Effects of Telmisartan with Hydrochlorothiazide versus Valsartan with Hydrochlorothiazide in Patients with Moderate-to-Severe Hypertension

**DOI:** 10.1155/2012/976828

**Published:** 2012-07-10

**Authors:** Ravi Marfatia, William B. White, Helmut Schumacher

**Affiliations:** ^1^Division of Hypertension and Clinical Pharmacology, Pat and Jim Calhoun Cardiology Center, University of Connecticut School of Medicine, Farmington, CT 06030, USA; ^2^Medical Data Services Department, Boehringer Ingelheim GmbH, Binger Straße 173, 5216 Ingelheim am Rhein, Germany

## Abstract

Combination therapy is recommended for patients with blood pressure (BP) significantly above goal by recent consensus guidelines around the globe. The use of angiotensin II receptor blockers (ARBs) alone or in combination with a thiazide diuretic is a preferred treatment strategy due to both efficacy and safety considerations. However, there are few data known about the benefits of ARB-diuretic combination therapy in patients with moderate-to-severe hypertension. We performed a subanalysis from two large clinical trials that compared the antihypertensive effects of telmisartan 80 mg versus valsartan 160 mg, both combined with hydrochlorothiazide (HCTZ) 25 mg in a subpopulation of 725 patients with moderate-to-severe hypertension (systolic BP SBP ≥ 160 mm Hg). Treatment with telmisartan-HCTZ induced significantly greater reductions in BP (−31.1/−18.3 mm Hg) than valsartan-HCTZ (−28.4/−16.3 mm Hg; SBP *P* = 0.0265, diastolic BP *P* = 0.0041). More patients receiving the telmisartan combination achieved a BP goal < 140/90 mm Hg than those receiving valsartan-HCTZ. There were similar safety and tolerability data for the two active treatment groups. These findings support the use of longer-acting ARBs combined with higher doses of thiazide diuretic to improve BP control in patients with moderate-to-severe hypertension.

## 1. Introduction 

The angiotensin II receptor blockers (ARBs) are effective antihypertensive agents with tolerability profiles similar to placebo [1–4]. The use of ARBs and/or angiotensin converting enzyme (ACE) inhibitors, alone or in combination with a calcium channel blocker or with a thiazide diuretic, has become the cornerstone of hypertension management [2, 5, 6]. A series of landmark clinical trials have demonstrated that ARBs reduce cardiovascular (CV) morbidity and mortality in a variety of types of hypertensive patients [7–13]. 

In seeking to improve blood pressure (BP) control, use of hydrochlorothiazide (HCTZ) at 25 mg rather than 12.5 mg in combination with ARBs and ACE inhibitors is recognized as an effective and well-tolerated strategy [1, 2, 14]. Two independent and identically designed trials were previously conducted to evaluate the benefits and safety of two single-pill combination (SPC) therapies: telmisartan 80 mg plus HCTZ 25 mg (T80/H25) and valsartan 160 mg plus HCTZ 25 mg (V160/H25) in patients with stages 1 and 2 hypertension [15, 16]. A pooled analysis of these two studies provided support for the use of ARBs with this higher 25 mg dose of thiazide diuretic; furthermore, the analysis demonstrated that T80/H25 resulted in greater reductions in clinic BP than V160/H25 [17]. 

As patients with moderate-to-severe hypertension have proportionally increased risk for CV morbidity and mortality, it is important to assess the magnitude of BP lowering with high doses of combination therapy in these hypertensive patients. In our pooled analysis of T80/H25 versus V160/H25, a substantial proportion of patients participating had stage (or grade) 2 hypertension [17]. The aim of the present analysis was to evaluate the efficacy and tolerability of both combination antihypertensive treatments in those patients who had moderate-to-severe hypertension, specifically defined as systolic BP (SBP) ≥ 160 mm Hg at baseline. 

## 2. Methods

### 2.1. Study Design 

The two studies had identical designs and were multicenter, double-blind, double-dummy, randomized, parallel-group studies that compared the efficacy and safety of T80/H25 versus V160/H25 or placebo. The methods and results for the entire population have been reported elsewhere [15–17]. 

The aim of the two trials was to determine whether T80/H25 mg administered once daily (o.d.) was superior to placebo administered o.d. and noninferior or superior to V160/H25 mg o.d. for the control of BP measured in the clinic following 8 weeks of treatment. A 3- to 4-week run-in period included a 1-week washout for patients currently receiving antihypertensive therapy, followed by a 2- to 3-week single-blind placebo period to establish baseline BP values. Eligible patients were then randomized to double-blind monotherapy treatment of telmisartan 80 mg, valsartan 160 mg, or placebo in a ratio of 4 : 4 : 1, respectively. After 2 weeks, all patients were uptitrated to combination treatment with T80/H25, V160/H25, or placebo, depending on their initial randomized treatment arm. 

### 2.2. Patient Population 

Men and women with average seated diastolic BP (DBP) of ≥95 mm Hg to ≤120 mm Hg at the end of the single-blind placebo treatment period were eligible for inclusion in both studies. The group of patients included in this current subanalysis had moderate-to-severe hypertension, defined as an average seated SBP of ≥160 mm Hg at baseline. Patients with stroke or myocardial infarction (MI) within the past 6 months, congestive heart failure, known or suspected secondary hypertension, poorly controlled diabetes mellitus, or chronic kidney failure were excluded from the studies.

### 2.3. Measurements of Efficacy and Safety Parameters 

Clinic BP was measured by study personnel using either a mercury column or aneroid manometry in the seated position. Three replicate measurements were averaged for analysis. Pulse rate was measured once in conjunction with BP at each study visit. Safety was assessed by evaluation of adverse events and vital signs at each study visit. All reported adverse events were categorized by body system and preferred term according to the Medical Dictionary for Regulatory Activities.

### 2.4. Statistical Analysis

Demographic, efficacy, and safety data for the subpopulation of moderate-to-severe hypertensive patients (i.e., patients with a baseline SBP of ≥160 mm Hg) were merged into a common database for analysis as described previously for the entire pooled population [17]. The primary endpoints for assessing efficacy were the changes from baseline to the end-of-study visit in clinic SBP and DBP, measured at the post dosing trough of study medication (23–26 h). In the case of patients withdrawing from the study prior to the completion of the 8-week treatment period, last-observation-carried-forward (LOCF) principles were utilized in those patients who had at least one set of BP measurements following titration to combination therapy. LOCF could only be used in those subjects who entered into the combination phase of the double-blind treatment period.

All analyses performed in the subpopulation of moderate-to-severe hypertensive patients were performed as *post hoc* assessments. Results of treatment comparisons are presented as two-sided *P* values. Mean changes in seated trough clinic BP per treatment group were adjusted for effects of baseline, study, gender, and race as previously described [17]. The impact of age, gender, and race on changes from baseline in SBP and DBP is also described. The probability of achieving BP goal (DBP < 90 mm Hg, SBP < 140 mm Hg) depending on baseline BP and treatment was calculated using logistic regression.

## 3. Results

### 3.1. Patient Enrolment and Disposition

A total of 2121 patients were randomized and received treatment (T80/H25, *n* = 942; V160/H25, *n* = 952; placebo, *n* = 227) across the two studies. Of these, 725 patients had moderate-to-severe hypertension at baseline (SBP ≥160 mm Hg) and were included in this subanalysis (T80/H25, *n* = 328; V160/H25, *n* = 317; placebo, *n* = 80).

### 3.2. Baseline Characteristics of Study Population 

Baseline characteristics of the subpopulation of patients with SBP ≥ 160 mm Hg are shown in Table 1. There were no differences in baseline characteristics observed among the three treatment arms. Mean age of the total population of patients with moderate-to-severe hypertension was 56.5, years and mean seated baseline BP was 167.7/103.3 mm Hg. Mean body mass index was 31.6 kg/m^2^, and the majority (79.9%) of patients were aged <65 years. Most of the patients were male (55.2%) and non-Black (72.8%). 

### 3.3. Changes in Clinic Trough (Approximately 24 h After-Dose) BP 

**Figure 1:**
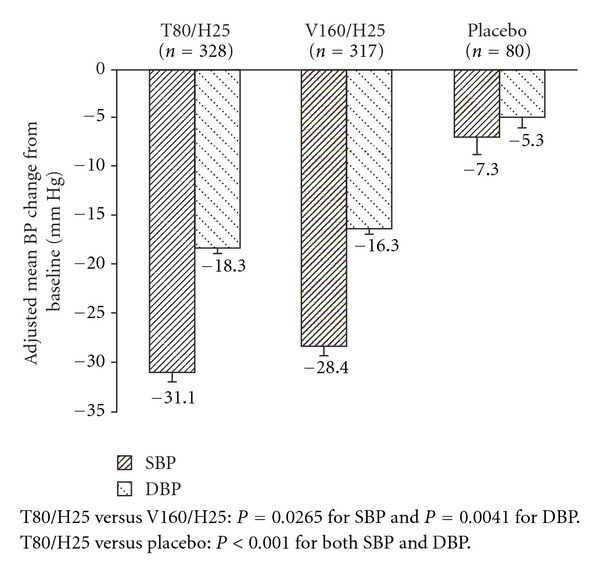
Change from baseline in SBP and DBP by treatment group. (T80/H25, telmisartan 80 mg/hydrochlorothiazide 25 mg: V160/H25, valsartan 160 mg/hydrochlorothiazide 25 mg).

**Figure 2:**
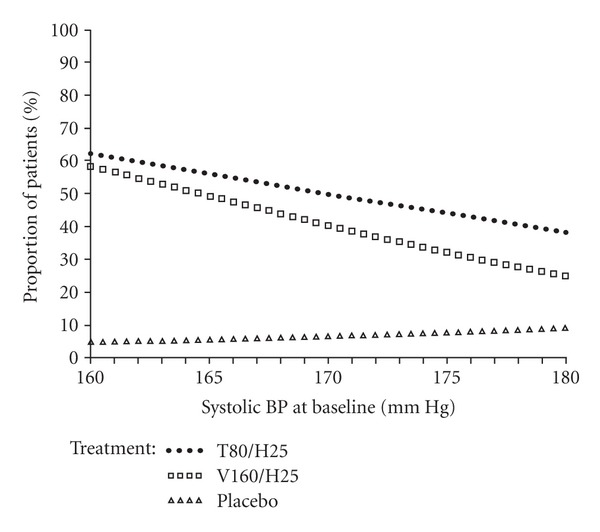
Proportion of patients achieving BP goal (<140/90 mm Hg), per treatment group, according to SBP at baseline (results from a logistic regression model allowing for treatment-by-baseline SBP interaction). T80/H25: telmisartan 80 mg/hydrochlorothiazide 25 mg. V160/H25:valsartan 160 mg/hydrochlorothiazide 25 mg.

The effects of the treatment on mean seated trough clinic BP among the subpopulation of patients with moderate-to-severe hypertension are illustrated in Figure 1 and tabulated in Table 2. BP was substantially lowered in both active treatment groups compared with placebo. Reduction in mean seated trough clinic BP with T80/H25 (−31.1/−18.3 mm Hg) was significantly greater than that with placebo (−7.3/−5.3 mm Hg; *P* < 0.0001 for both SBP and DBP). Treatment with V160/H25 induced reductions in mean seated trough clinic BP of –28.4/−16.3 mm Hg. Treatment with T80/H25 was associated with a significantly greater mean reduction in BP compared with V160/H25 for both SBP (adjusted mean difference −2.7 mm Hg; 95% confidence interval (CI), −5.1, −0.3; *P* = 0.0265) and DBP (adjusted mean difference −2.0 mm Hg; 95% CI, −3.4, −0.6; *P* = 0.0041). The probability of achieving BP goal (<140/90 mm Hg) was dependent on baseline SBP and differed between treatment arms (Figure 2). Averaged over the complete range of baseline SBP, the probability was 4% with placebo, 52% with T80/H25, and 43% with V160/H25 (odds ratio comparing T80/H25 and V160/H25 was 1.47; 95% CI, 1.06, 2.02, *P* = 0.0194).

### 3.4. Impact of Age, Gender, and Race on Changes in BP 

**Figure 3:**
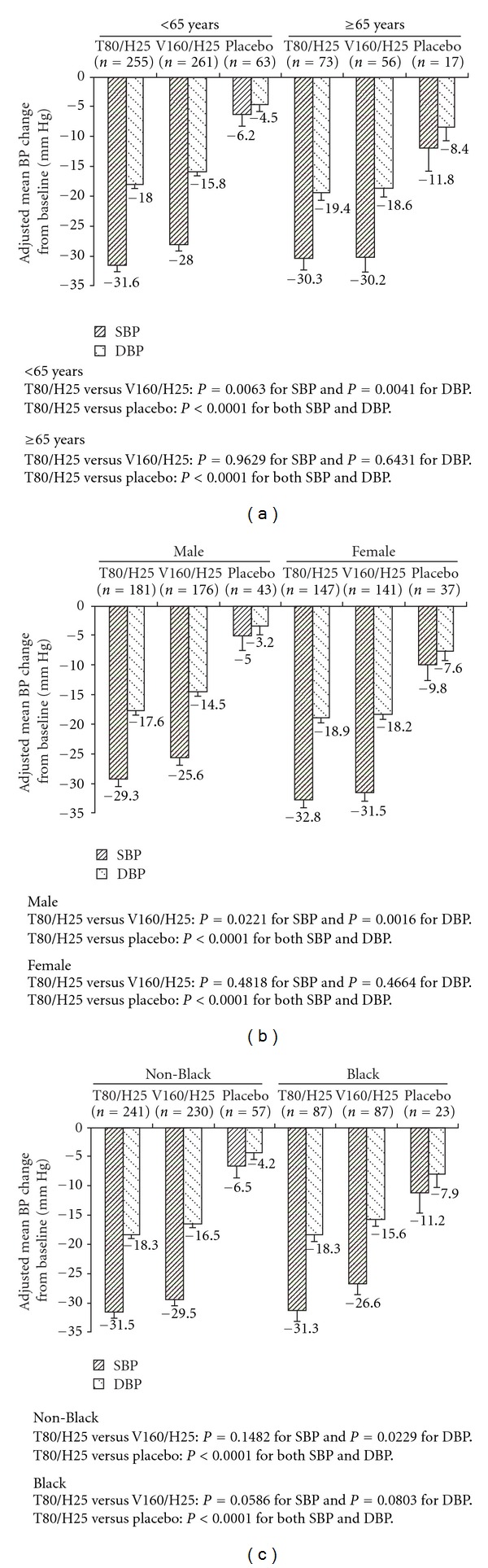
Impact of age (a), gender (b), and race (c) on changes from baseline in BP by treatment group. T80/H25, telmisartan 80 mg/hydrochlorothiazide 25 mg: V160/H25, valsartan 160 mg/hydrochlorothiazide 25 mg.

The impact of age (<65 and ≥65 years) on reductions in BP is shown in Figure 3(a). In patients aged <65 years, T80/H25 resulted in significantly greater BP reductions from baseline (adjusted mean SBP/DBP change −31.6/−18.0 mm Hg) than V160/H25 (adjusted mean SBP/DBP change −28.0/−15.8 mm Hg; *P* = 0.0063 for difference in SBP and *P* = 0.0041 for DBP). No significant differences in BP change were observed between the active treatment groups in patients aged ≥65 years (−30.3/−19.4 mm Hg for T80/H25 versus −30.2/−18.6 mm Hg for V160/H25).

The impact of gender on reductions in BP is shown in Figure 3(b). In male patients, T80/H25 resulted in significantly greater BP reductions from baseline (adjusted mean SBP/DBP change −29.3/−17.6 mm Hg) than V160/H25 (adjusted mean SBP/DBP change −25.6/−14.5 mm Hg; *P* = 0.0221 for difference in SBP and *P* = 0.0016 for DBP). No significant differences in BP change were observed between the active treatment groups in female patients (−32.8/−18.9 mm Hg for T80/H25 versus −31.5/−18.2 mm Hg for V160/H25). Greater changes from baseline in SBP and DBP were seen in female patients compared with males across the study, and this gender difference was consistent across the three treatment groups. 

The impact of race (non-Black and Black) on changes in BP is shown in Figure 3(c). Among non-Black patients, T80/H25 resulted in significantly greater DBP reductions from baseline (adjusted mean DBP change −18.3 mm Hg) than V160/H25 (adjusted mean DBP change −16.5 mm Hg; *P* = 0.0229). No significant differences in SBP change were observed between the active treatment groups in non-Black patients (−31.5 mm Hg for T80/H25 versus −29.5 mm Hg for V160/H25). Among Black patients, there were no significant differences in BP change between the active treatment groups (adjusted mean SBP/DBP change –31.3/–18.3 mm Hg for T80/H25 versus –26.6/−15.6 mm Hg for V160/H25).

### 3.5. Adverse Events 

Of the patients randomized to the study who received at least one dose of study medication and were within the subpopulation of patients with moderate-to-severe hypertension (*n* = 751), 204 (27.2%) had at least one adverse event: 94 (27.9%) in the T80/H25 arm, 73 (22.3%) in the V160/H25 arm, and 37 (42.5%) in the placebo arm. Three patients in the T80/H25 arm (0.9%) experienced serious adverse events, compared with one (0.3%) in the V160/H25 arm and none in the placebo arm. None of the patients died. The rate and type of adverse events reported among this subpopulation of patients with moderate-to-severe hypertension were similar to those previously reported for the entire study population [15–17]. The most common adverse events (occurring in >2% of patients with moderate-to-severe hypertension) are provided in Table 3.

## 4. Discussion

This new analysis of data in the moderate-to-severe hypertensive patients from our two identically designed trials has shown that treatment with T80/H25 lowered SBP and DBP to a significantly greater extent than treatment with V160/H25. As expected, both active treatments lowered BP to a significantly greater extent than placebo. These data build on earlier studies that demonstrated additive and dose-proportionate decreases in BP with telmisartan/HCTZ combinations, and which highlight that the greatest reductions in BP are achieved in patients with the highest pretreatment BP [3].

Our analysis shows that large reductions from baseline in BP in those patients whose BP is substantially above clinical targets can be achieved with the combination of an ARB and higher-dose diuretic SPC therapies. After 8 weeks of active treatment, a final mean SBP/DBP <140/90 mm Hg occurred in approximately half of the patients with moderate-to-severe hypertension, representing a clinically important achievement in this high-risk patient group. Furthermore, this analysis showed that different ARBs within the SPC therapy had an impact on BP outcomes and targets. Treatment with T80/H25 was significantly more effective than V160/H25 in terms of the proportion of patients who achieved a BP goal of <140/90 mm Hg.

The results of the analysis herein are comparable to previous studies that have assessed the dose response to valsartan in combination with HCTZ. For example, in a randomized controlled trial reported by Benz et al. [18], V160/H25 mg was shown to lower BP by 22/15 mm Hg following 8 weeks of treatment, as compared with V160/H12.5 mg, which lowered BP by 18/14 mm Hg. A study by Trenkwalder and colleagues also demonstrated that patients not controlled on V160/H12.5 mg could achieve an additional 8.4/8.3 mm Hg reduction in BP when the diuretic dose was increased to 25 mg [19]. These BP-lowering results are similar to those reported for the overall population of patients pooled from our two studies (V160/H25 lowered BP by 22.3/16.8 mm Hg) [17] and to the reductions reported in the current analysis (Figure 1; Table 1), in which V160/H25 lowered BP by 28.4/16.3 mm Hg and T80/H25 lowered BP by 31.1/18.3 mm Hg at Week 8 in patients with moderate-to-severe hypertension. 

In previous studies comparing telmisartan with valsartan as monotherapy in similar doses, telmisartan was shown to induce more sustained BP control, with both greater 24 h BP reductions and significantly greater reductions in BP during the last 6 h of the dosing period than valsartan [20, 21]. This may be partly explained by the pharmacokinetic profile of telmisartan, which has a longer half-life (24 h) compared with valsartan (7 h) [22, 23].

In the studies and analysis reported here, despite the potential for “equalizing” the BP-reducing effects of these two ARBs by combining treatments with 25 mg thiazide diuretic, differences between telmisartan and valsartan were preserved, with significantly greater reductions in BP noted in the patients treated with the telmisartan/HCTZ combination. The analysis of patients with moderate-to-severe hypertension also found that the BP-lowering efficacy of T80/H25 was consistent across age, gender, and race subgroups. In contrast, the effect of valsartan was more variable. Furthermore, T80/H25 always showed larger BP reductions than V160/H25 across all subgroups, although these differences were not always significant. These results support the use of T80/H25 in patients with moderate-to-severe hypertension, regardless of age, gender, and race. 

The magnitude of the differences in BP reductions observed in our analysis in the moderate-to-severe hypertensive patient population between treatment groups is clinically relevant. Mean reductions in BP were greater than 20/10 mm Hg for the SPC therapies, and the differences between T80/H25 and V160/H25 were also of clinical relevance. In a meta-analysis of one million adults from 61 prospective studies, the relationship between BP reduction and CV morbidity and mortality events supports that a reduction in SBP of just 2 mm Hg would provide a 10% reduction in stroke mortality and 7% lower mortality from US spelling-ischaemic heart disease and other vascular deaths [24]. In an earlier study that assessed observational data from two large population cohorts, a 2 mm Hg reduction in DBP was shown to be associated with a 9% reduction in risk of coronary heart disease and a 15% reduction in stroke [25]. 

There is a good body of evidence showing that antihypertensive treatment involving ARBs can reduce CV morbidity and mortality in different types of at-risk hypertensive patients, such as those with additional CV risk factors [8, 9], patients with diabetes and documented target organ damage or a history of stroke and MI [13], and those with heart failure [10–12] and end-stage renal disease [7]. The clinical importance of using antihypertensive combinations that offer optimal BP reductions has also been highlighted in the findings of a number of large-scale intervention studies. For example, the Antihypertensive and Lipid-Lowering Treatment to Prevent Heart Attack Trial (ALLHAT) [26] and Valsartan Antihypertensive Long-term Use Evaluation (VALUE) trial [9] both demonstrated that when one pharmacological regimen induces greater reductions in BP than another, this may have important clinical implications in terms of reduction in CV and cerebrovascular morbidity, even during a treatment period of less than 1 year. 

More recently, the Avoiding Cardiovascular Events through Combining Therapy in Patients Living with Systolic Hypertension (ACCOMPLISH) trial in patients at high risk for CV events, compared two treatment combinations that differed by just 1 mm Hg in terms of mean SBP and DBP and reported that the more effective BP-lowering regimen was associated with a significant reduction in a primary composite endpoint of death from CV causes, nonfatal MI, nonfatal stroke, hospitalization for angina, resuscitation after sudden cardiac arrest, and coronary revascularizsation [6].

## 5. Conclusion

This pooled analysis confirmed that SPC therapy with T80/H25 o.d. reduced both SBP and DBP to a greater extent than V160/H25 in patients with moderate-to-severe hypertension, defined as SBP ≥ 160 mm Hg at baseline. The superior antihypertensive efficacy of the telmisartan/HCTZ combination observed may reflect the longer half-life of telmisartan of 24 h compared with 7–9 h for valsartan. Analysis of pooled data therefore demonstrates that, in patients with moderate-to-severe hypertension, T80/H25 provides superior efficacy to V160/25, with a high rate of BP goal achievement and similar levels of tolerability. These findings support the use of long-acting ARBs such as telmisartan in combination with higher doses of thiazide diuretic (25 mg) to provide improved BP control in the more severely hypertensive patient. 

## Figures and Tables

**Table 1:** Baseline characteristics.

	T80/H25	V160/H25	Placebo
*N*	328	317	80
Age, years (SD)	57.0 (10.7)	56.0 (9.7)	56.4 (10.6)
Age group, *N* (%)			
<65 years	255 (77.7)	261 (82.3)	63 (78.8)
≥65 years	73 (22.3)	56 (17.7)	17 (21.3)
Gender, *N* (%)			
Male	181 (55.2)	176 (55.5)	43 (53.8)
Female	147 (44.8)	141 (44.5)	37 (46.3)
Race, *N* (%)			
Non-Black	241 (73.5)	230 (72.6)	57 (71.3)
Black	87 (26.5)	87 (27.4)	23 (28.8)
BMI, kg/m^2^ (SD)	31.4 (6.1)	31.8 (6.8)	31.5 (6.2)
SBP, mm Hg (SD)	167.6 (5.4)	168.3 (5.9)	167.1 (5.8)
DBP, mm Hg (SD)	103.2 (4.4)	103.4 (4.8)	103.3 (4.0)
Pulse rate, beats/min (SD)	75.7 (9.9)	75.0 (9.4)	74.5 (8.8)

BMI: body mass index; DBP: diastolic blood pressure; SBP: systolic blood pressure; SD: standard deviation; T80/H25: telmisartan 80 mg/hydrochlorothiazide 25 mg; V160/H25: valsartan 160 mg/hydrochlorothiazide 25 mg.

**Table 2:** Mean seated clinic trough BP and changes from baseline by treatment group.

	T80/H25 (*n* = 328)	V160/H25 (*n* = 317)	Placebo (*n* = 80)
SBP, mm Hg			
Baseline (SD)	167.6 (5.4)	168.3 (5.8)	167.1 (5.9)
End of study (SD)	136.6 (15.3)	139.75 (16.4)	160.2 (14.9)
Change from baseline (SD)	−31.1 (15.1)	−28.6 (15.9)	−7.0 (14.3)
Adjusted^a^ change from baseline (SE)	−31.1 (0.9)	−28.4 (0.9)	−7.3 (1.7)
Comparison to T80/H25 (95% CI)		−2.7 (−5.1, −0.3) *P* = 0.0265	−23.8 (−27.5, −20.0) *P* < 0.0001
DBP, mm Hg			
Baseline (SD)	103.2 (4.4)	103.4 (4.8)	103.4 (4.1)
End of study (SD)	85.1 (9.4)	87.3 (10.4)	98.3 (10.1)
Change from baseline (SD)	−18.1 (8.7)	−16.1 (9.3)	−5.0 (9.5)
Adjusted^a^ change from baseline (SE)	−18.3 (0.52)	−16.3 (0.5)	−5.3 (1.0)
Comparison to T80/H25 (95% CI)		−2.0 (−3.4, −0.6) *P* = 0.0041	−13.0 (−15.2, −10.8) *P* < 0.0001

BP: blood pressure; CI: confidence interval; DBP: diastolic blood pressure; SBP: systolic blood pressure; SD: standard deviation; SE: standard error; T80/H25: telmisartan 80 mg/hydrochlorothiazide 25 mg; V160/H25: valsartan 160 mg/hydrochlorothiazide 25 mg.

^a^Adjusted for effects of baseline, study, gender, and race.

**Table 3:** Adverse events with incidence ≥2%.

	T80/H25 (*n* = 337)	V160/H25 (*n* = 327)	Placebo (*n* = 87)
Patients with any adverse event, *n* (%)	94 (27.9)	73 (22.3)	37 (42.5)
Upper respiratory tract infection, *n* (%)	6 (1.8)	5 (1.5)	3 (3.4)
Sinusitis, *n* (%)	2 (0.6)	4 (1.2)	2 (2.3)
Headache, *n* (%)	8 (2.4)	7 (2.1)	4 (4.6)
Dizziness, *n* (%)	7 (2.1)	7 (2.1)	3 (3.4)
US spelling-diarrhea *n* (%)	5 (1.5)	2 (0.6)	3 (3.4)
Dry mouth, *n* (%)	1 (0.3)	1 (0.3)	2 (2.3)
Vomiting, *n* (%)	—	2 (0.6)	2 (2.3)
Muscle spasm, *n* (%)	3 (0.9)	5 (1.5)	2 (2.3)
Hypertension, *n* (%)	—	—	5 (5.7)
Fatigue, *n* (%)	6 (1.8)	2 (0.6)	2 (2.3)

T80/H25: telmisartan 80 mg/hydrochlorothiazide 25 mg; V160/H25: valsartan 80 mg/hydrochlorothiazide 25 mg.
